# A New Antifungal-Loaded Sol-Gel Can Prevent *Candida albicans* Prosthetic Joint Infection

**DOI:** 10.3390/antibiotics10060711

**Published:** 2021-06-12

**Authors:** Hugo Garlito-Díaz, Jaime Esteban, Aranzazu Mediero, Rafael Alfredo Carias-Cálix, Beatriz Toirac, Francisca Mulero, Víctor Faus-Rodrigo, Antonia Jiménez-Morales, Emilio Calvo, John Jairo Aguilera-Correa

**Affiliations:** 1 Department of Orthopaedic Surgery, Infanta Elena University Hospital, 28342 Valdemoro, Spain; hugo.garlito@quironsalud.es (H.G.-D.); ecalvo@fjd.es (E.C.); 2 Department of Orthopaedic Surgery, Fundación Jiménez Diaz University Hospital, 28040 Madrid, Spain; 3 Department of Clinical Microbiology, IIS-Fundación Jiménez Diaz, UAM, 28040 Madrid, Spain; 4 Bone and Joint Research Unit, IIS-Fundación Jiménez Diaz, UAM, 28040 Madrid, Spain; aranzazu.mediero@quironsalud.es; 5 Pathology Department, Fundación Jiménez Diaz University Hospital, UAM, 28040 Madrid, Spain; Rafael.carias@quironsalud.es; 6 Materials Science and Engineering Department, University Carlos III of Madrid, 28040 Madrid, Spain; btoirac@ing.uc3m.es (B.T.); toni@ing.uc3m.es (A.J.-M.); 7 Molecular Imaging Unit, Spanish National Cancer Research Centre (CNIO), 28040 Madrid, Spain; fmulero@cnio.es; 8 Experimental Surgery and Animal Research Service, IIS-Fundación Jiménez Diaz, UAM, 28040 Madrid, Spain; victor.faus@quironsalud.es; 9 Álvaro Alonso Barba Technological Institute of Chemistry and Materials, Carlos III University of Madrid, 28040 Madrid, Spain

**Keywords:** sol-gel, anidulafungin, prosthetic joint infection, *Candida albicans*

## Abstract

Fungal PJI is one of the most feared complications after arthroplasty. Although a rare finding, its high associated morbidity and mortality makes it an important object of study. The most frequent species causing fungal PJI is *C. albicans*. New technology to treat this type of PJI involves organic–inorganic sol-gels loaded with antifungals, as proposed in this study, in which anidulafungin is associated with organophosphates. This study aimed to evaluate the efficacy of an anidulafungin-loaded organic–inorganic sol-gel in preventing prosthetic joint infection (PJI), caused by *Candida albicans* using an in vivo murine model that evaluates many different variables. Fifty percent (3/6) of mice in the *C. albicans*-infected, non-coated, chemical-polished (CP)-implant group had positive culture and 100% of the animals in the *C. albicans*-infected, anidulafungin-loaded, sol-gel coated (CP + A)-implant group had a negative culture (0/6) (*p* = 0.023). Taking the microbiology and pathology results into account, 54.5% (6/11) of *C. albicans*-infected CP-implant mice were diagnosed with a PJI, whilst only 9.1% (1/11) of *C. albicans*-infected CP + A-implant mice were PJI-positive (*p* = 0.011). No differences were observed between the bone mineral content and bone mineral density of noninfected CP and noninfected CP + A (*p* = 0.835, and *p* = 0.181, respectively). No histological or histochemical differences were found in the tissue area occupied by the implant among CP and CP + A. Only 2 of the 6 behavioural variables evaluated exhibited changes during the study: limping and piloerection. In conclusion, the anidulafungin-loaded sol-gel coating showed an excellent antifungal response in vivo and can prevent PJI due to *C. albicans* in this experimental model.

## 1. Introduction

Osteoarthritis is one of the most common musculoskeletal diseases worldwide, and is the most well-known cause of disability among elderly people [1]. The social and economic burden of osteoarthritis-related loss of work is also high [2]. Joint replacement is a treatment approach that improves quality of life in many individuals worldwide. Although already used routinely, prosthesis implantation is likely to continue to rise in the coming years [3,4]. The primary cause of device failure is prosthetic joint infection (PJI), a disease involving joint prosthesis and nearby tissue. Advances in the study of the transmission, diagnosis, and treatment of PJI over the last 25 years have led to an improvement in outcomes following this difficult complication. PJI occurs rarely (1–2% of all cases), although its effects are often devastating due to the high associated morbidity and substantial costs [2,5,6]. Additionally, the economic burden of PJI is expected to rise in the coming years with increasing life expectancy and the resulting increase in the number of patients undergoing arthroplasty replacements [7].

The most frequently isolated pathogens from PJI are Gram-positive bacteria, especially *Staphylococcus* species, and Gram-negative microorganisms. Nevertheless, other microorganisms, such as fungi, can also cause PJI, particularly *Candida* species [8,9,10,11,12,13]. *C. albicans* is the most frequent pathogen isolated, followed by *C. parapsilosis* [10]. Most fungal PJIs present with an insidious, chronic clinical course and are associated with risk factors such as advanced age, previous infection with *Candida*, prior antimicrobial use, multiple surgeries on the joint, immunosuppression, and diabetes [10,14,15,16]. Despite being a rare infection (<1%), up to a quarter of cases can progress to candidemia, which carries an associated mortality of up to 40% [17]. This type of PJI poses a challenge for clinicians and requires a multidisciplinary approach, including systemic antibiotics, local therapies, and surgery [18,19,20]. The systemic and prophylactic treatment of PJIs may be ineffective, as antimicrobials are incapable of reaching the prosthesis–tissue interface due to the continued presence of necrotic and/or avascular tissue after surgery [21].

To address this problem, local antibiotic therapy was proposed as an alternative and/or adjuvant to systemic prophylaxis or treatment, preventing systemic toxicity and favouring drug release directly within the implant site [22]. Organic–inorganic sol-gels loaded with antifungals were used in this approach. Recently, the incorporation of organophosphate [tris(trimethylsilyl) phosphite] in this sol-gel, made of two silanes (3-methacryloxypropyl trimethoxysilane and 2-tetramethyl orthosilicate), has been shown to enhance the adhesion of sol-gel on metallic surfaces and increase cell proliferation [23]. Recently, some studies have reported the excellent biosafety and bactericidal capacity of these materials, showing that they completely inhibit the formation of biofilm by *S. epidermidis* in venous catheters without deleterious procoagulant effects in the animal model [24]. Furthermore, new studies show that sol-gel coatings loaded with fluconazole can prevent and locally treat yeast PJI, specifically those caused by the *Candida* species [25].

This study aimed to evaluate the efficacy of an anidulafungin-loaded, organic–inorganic sol-gel in preventing PJI caused by *C. albicans* using an in vivo murine model.

## 2. Results

### 2.1. Animal Monitoring

**Figure 1:**
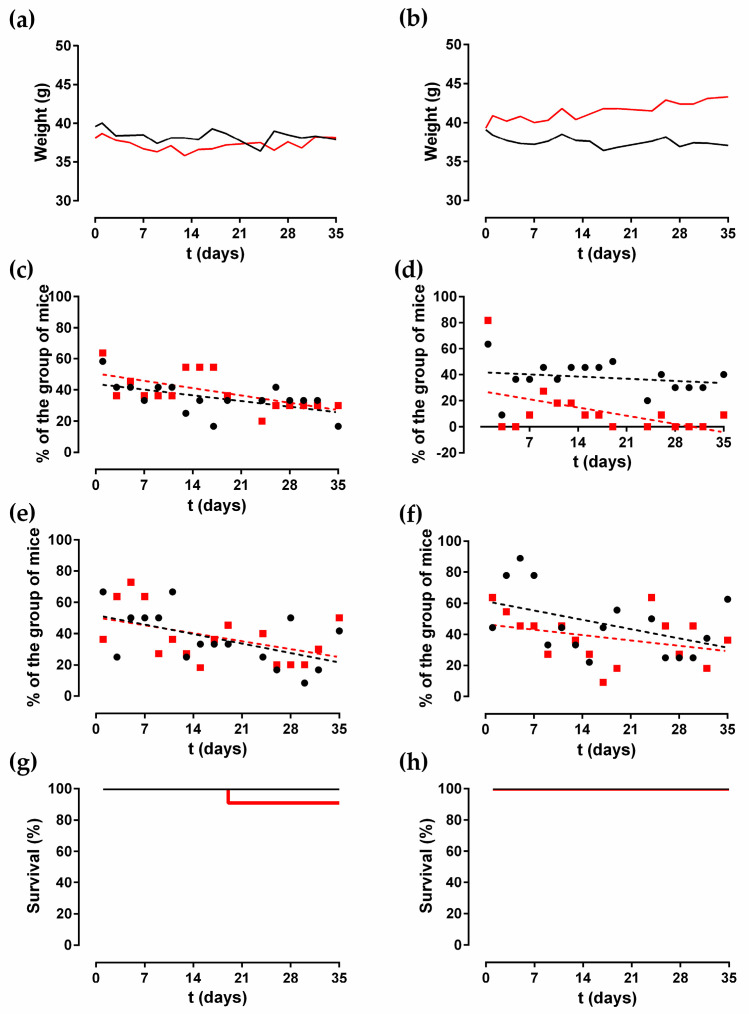
Median weight (**a**,**b**), limping (**c**,**d**), piloerection (**e**,**f**), and survival (**g**,**h**) in different noninfected groups (black) and in the Cal 35-infected group (red) with insertion of CP (left column) and CP + A implant (right column) over time.

The median weight of the mice over time by group is shown in Figure 1a,b. Only the group of mice infected with *C. albicans* (Cal 35) after the insertion of an anidulafungin-loaded, coated, chemically polished (CP + A) implant showed a significant increase in weight of 44.23 mg per day (*p* = 0.0189). The weight of the remaining groups showed no change over time (*p* > 0.05).

Only two of the six behavioural variables evaluated exhibited changes during the study: limping and piloerection. In the groups with uncoated CP implants, limping decreased significantly over time in both noninfected and Cal35-infected groups (*p* = 0.025, and *p* = 0.026, respectively). The slope of the limping was higher in the Cal35-infected group than in the noninfected one: −0.6694% per day versus −0.5198% per day, respectively (Figure 1c). In both groups of mice with a CP + A implant, limping stayed constant over time (*p* > 0.05) (Figure 1d).

In the groups of animals with CP implants, only the noninfected group showed a significant decrease in piloerection over time (*p* = 0.031), with a slope of −0.8635% per day (Figure 1e). In the mice with an inserted CP + A-implant, piloerection stayed constant over time in both groups (Figure 1f).

**Figure 2:**
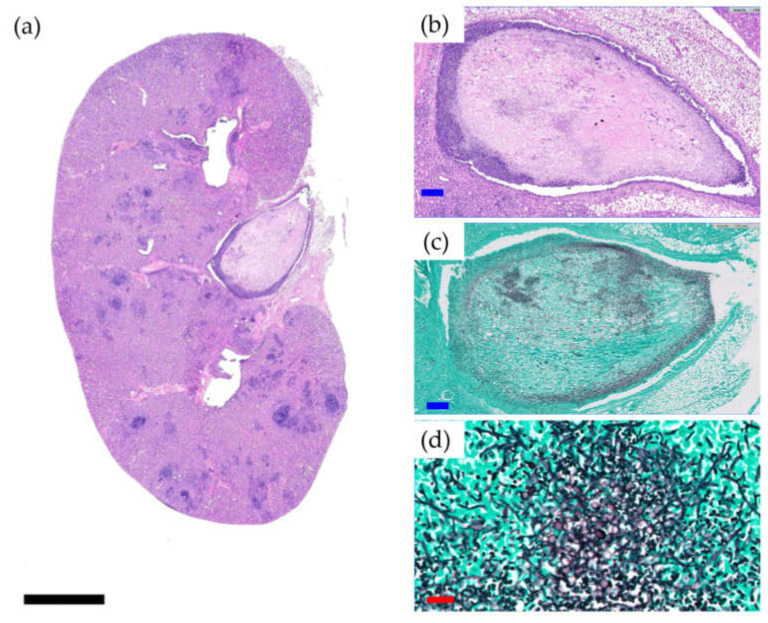
Histological section of the kidney of a mouse belonging to the Cal35-infected CP-implant group that died of candidemia (**a**) and histological sections at higher magnifications in haematoxylin and eosin staining (**b**) and Groccot’s stain (**c**,**d**). Black, blue, and red bars represent 2 mm, 50 µm, and 20 µm, respectively.

**Figure 3:**
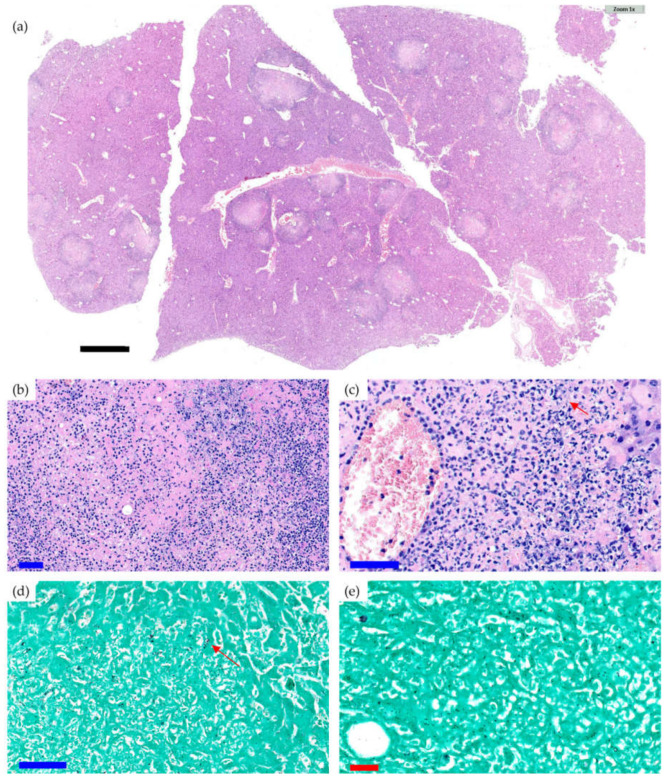
Histological section of the liver of a mouse of the CP Cal35 group that died of CMV infection (**a**) and histological sections with a greater increase in H&E (**b**,**c**) and Groccot’s silver stain (**d**,**e**). Black, blue, and red bars represent 2 mm, 50 µm, and 20 µm, respectively. The liver of the animal showed parenchyma with foci of necrosis and a polymorphonuclear-type inflammatory infiltration accompanied by occasional Grocott-positive intracellular inclusions inside some hepatocytes, which was compatible with a cytomegalovirus infection. An acute necrotising inflammatory reaction was detected around the central veins.

Survival was significantly lower in the Cal35-infected group with CP implants than in the noninfected group as of day 19 (*p* = 0.002) (Figure 1g). Only one mouse (9.1%) in the Cal35-infected group with CP implants died of candidemia (Figure 2). Only one mouse in the Cal35-infected group with CP + A implants died because of a Cytomegalovirus infection (Figure 3); for this reason, this mouse was withdrawn from the survival analysis. Taking this into account, no survival differences were detected between CP + A-implants group and Cal35-infected mice with the CP + A-implants group (Figure 1h).

### 2.2. Microbiological and Pathological Results

**Figure 4:**
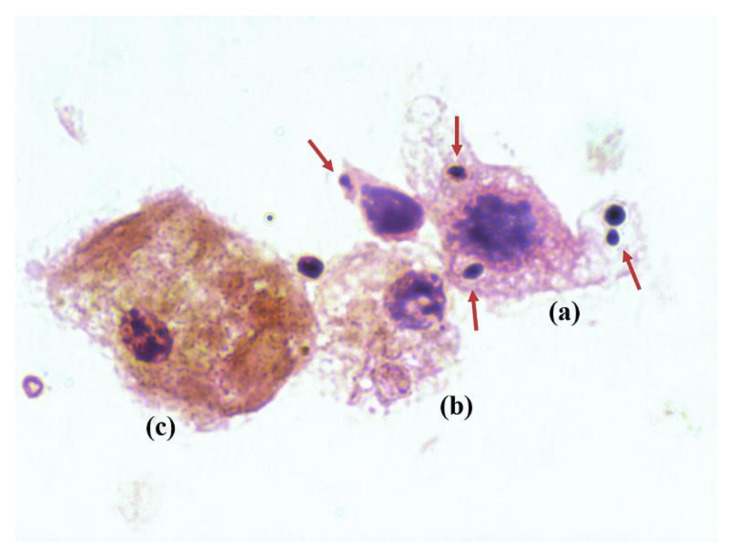
Cytological image of a Gram stain showing a macrophage (**a**,**b**) phagocytizing multiple yeasts (red arrows), and a myocyte (**c**).

The femur culture of the noninfected groups was negative for all of mice. Three of the 11 stamps from Cal35-infected CP-implant mice revealed the presence of yeast in the synovial fluid on Gram staining (Figure 4). All of the stamps from the Cal35-infected mice with CP + A-implants were negative. Each Cal-35-infected group composed of 11 mice was divided into two subgroups: six animals were used for microbiological studies and five for pathological studies.

**Figure 5:**
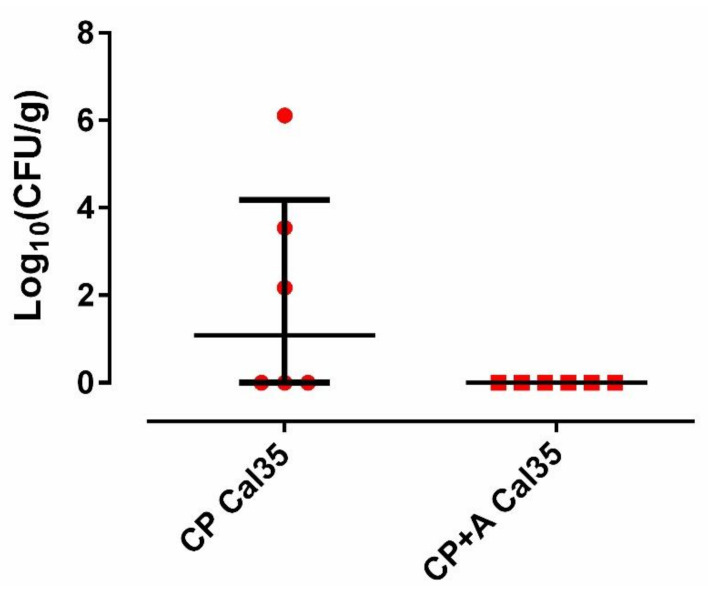
Quantity of yeast per gram of femur from each group of mice.

Fifty percent (3/6; 95%CI: 0.099–0.900) of mice in the Cal 35-infected group of mice with CP implants had positive culture, whilst 100% of the Cal 35-infected animals with CP + A-implants had a negative culture (0/6) (*p* = 0.023). No statistically significant difference was observed in the quantity of yeast per gram of femur between the 2 Cal35-infected groups (*p* = 0.091) (Figure 5). The mouse that died in the Cal35-infected group with CP implants had granulomas in both kidneys and a concentration of 4.02 log_10_ (colony-forming units per femur, CFU/femur) on the outside of the operated femur, 5.51 log_10_(CFU/g) in the operated femur, 5.47 log_10_(CFU/g) in the kidney, and 25.5 CFU/cm^2^ of the implant surface. The renal parenchyma showed extensive Grocott-positive fungal involvement accompanied by intense acute polymorphonuclear-type inflammation, which presented a patchy distribution pattern affecting both the renal cortex and medulla and the pyelocaliceal system (Figure 2).

**Figure 6:**
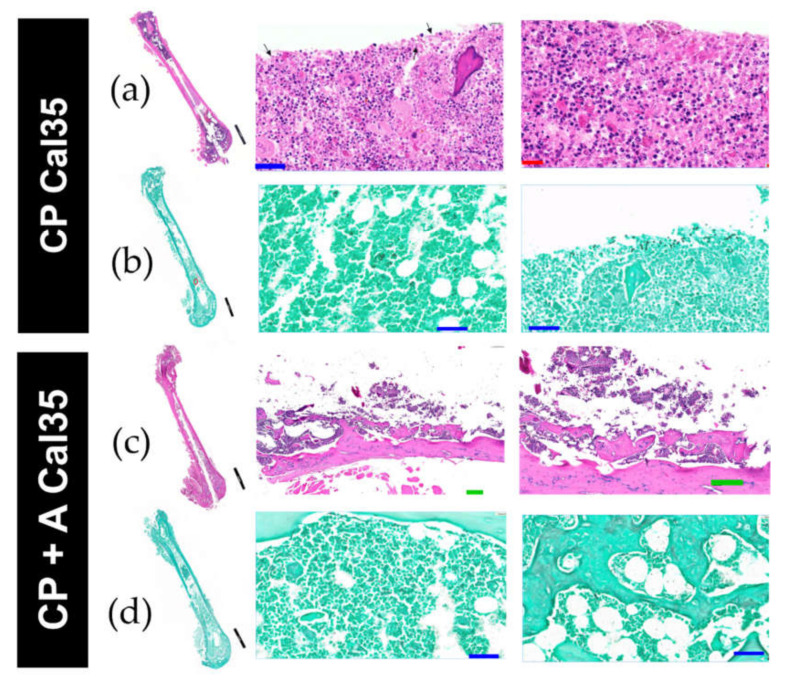
Histological images with H&E stain (**a**,**c**) and Grocott’s stain (**b**,**d**) of Cal-35–infected mice with implant without CP coating (**a**,**b**) and mice infected with implant and anidulafungin coating CP + A (**c**,**d**). The black, green, blue, and red bars represent 2 mm, 200 µm, 50 µm, and 20 µm, respectively.

In the Cal35-infected group with CP implants, acute osteomyelitis was observed in four of the five femurs (Figure 6a); no chronic osteomyelitis was diagnosed, and the presence of yeast was also detected in four of the five femurs on Grocott’s silver staining (Figure 6b). In the Cal35-infected group with CP + A-implants, acute osteomyelitis was observed in two of the five femurs (Figure 6c,d), chronic osteomyelitis was diagnosed in two of the five femurs, and presence of yeast was detected in only one of the five femurs following Grocott´s silver staining; the latter also showed the presence of acute osteomyelitis. Only one mouse (9.1%) in the noninfected group of animals with a CP + A-implant died (Figure 3). The deceased mouse from this group showed signs of having had diarrhoea, enteritis, and hepatomegaly. Furthermore, when the operated femur, both kidneys, and a piece of the liver were sent for microbiological study, no growth in aerobic or facultative anaerobic bacteria or fungi was detected.

Taking both the microbiology results and pathology results into account, 54.5% of the Cal35-infected mice with CP implants were diagnosed with a PJI, whilst only 9.1% of the Cal35-infected mice with CP + A-implants were PJI-positive. Therefore, the PJI positivity was significantly higher in the Cal35-infected CP-implant group than in the Cal-35 CP + A-implant group (*p* = 0.011).

The presence of round or ovoid structures accompanied by signs of germination was noteworthy, as was as the presence of other septate structures corresponding to pseudo-hyphae and hyphae, visible with Grocott’s stain. No other infectious agents were observed in the samples studied.

### 2.3. Microcomputed Tomography and Bone Histology

**Figure 7:**
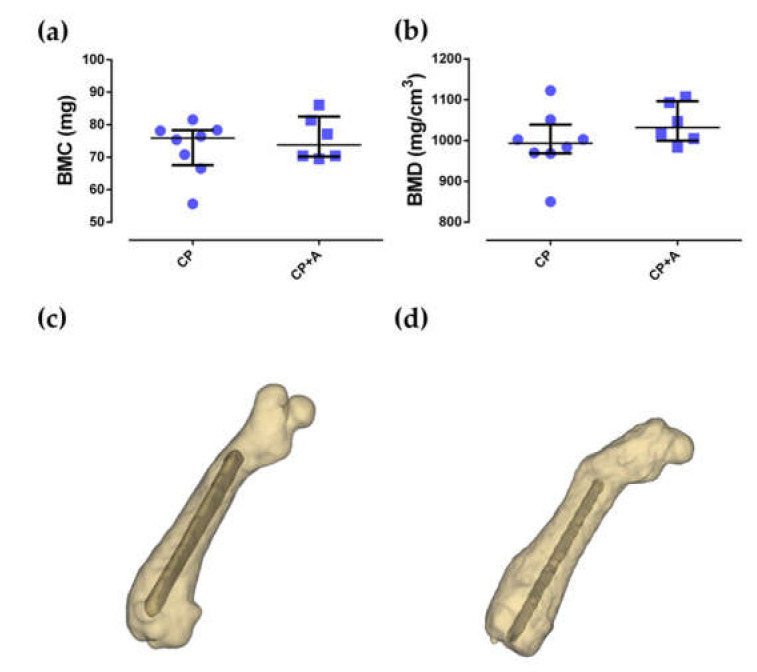
Bone mineral content (BMC) (**a**) and bone mineral density (BMD) **(b**) and their three-dimensional reconstructions of a representative sample of the CP group (**c**) and CP + A group (**d**).

No differences were observed between the bone mineral content (BMC) and bone mineral density (BMD) of the groups of mice with CP- and CP + A-implants (*p* = 0.835, and *p* = 0.181, respectively). The BMD results were perfectly comparable as there were no differences in BMC between the compared groups (Figure 7).

**Figure 8:**
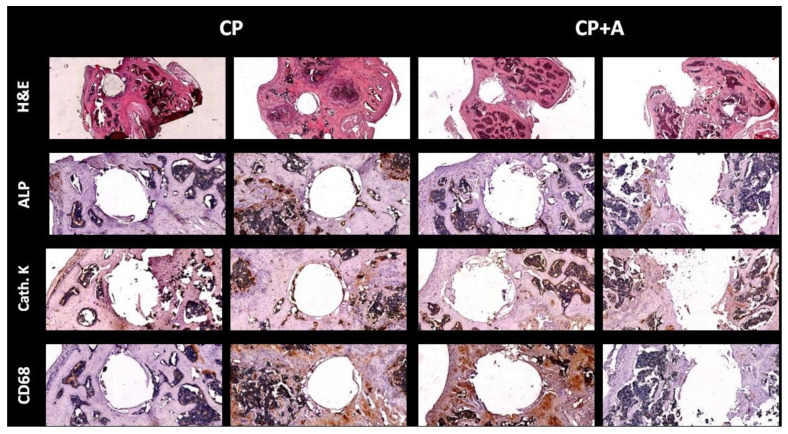
Immunohistochemistry for markers of different bone cells. Long bones were processed and immunohistology staining was carried out. Representative images stained for haematoxylin-eosin (H&E), tartrate-resistant acid phosphatase (TRAP) staining, cathepsin K (cath. K), alkaline phosphatase (ALP), and macrophages (cluster of differentiation 68, CD68). H&E images were taken at 4× magnification. All immunostaining images were taken at 10× magnification.

Hematoxilin-eosin staining showed no differences in tissue in the area occupied by the implant among the mice with CP- and CP + A-implants. When bone markers were analysed in the defect area, no changes were observed among the different animals, in alkaline phosphatase (ALP) staining, Cathepsin K or cluster of differentiation 68 (CD68) (Figure 8).

The viable medullary zones are of a habitual trilinear aspect and were arranged in the peripheral ends of the bone (epiphysis).

## 3. Discussion

In this study, we demonstrate the in vivo efficacy of anidulafungin-loaded sol-gel to prevent PJI caused by *C. albicans* using a murine model. We describe a novel approach to sol-gel technology applied to Ti materials using anidulafungin to locally prevent the development of yeast biofilms.

The most frequent clinical manifestations of PJI are pain, joint swelling or effusion, erythema or warmth around the joint, fever, drainage, and the presence of a sinus tract connecting to the prosthesis [2]. In our in vivo model, joint pain was evaluated by monitoring mice weight, limping, and piloerection. Weight remained constant over time in all groups, except the Cal35-infected CP + A-implant group, where the mice increased in weight over time. This discrepancy in weight is uncertain, but it could be attributed to the effect of dexamethasone, which has been shown to both increase [26] and reduce mouse weight [27,28]. Moreover, it is known that enrofloxacin does not impede weight gain [29]. Infection seems to decrease limping more significantly, as can be seen in the CP-implant groups. *C. albicans*-caused infections were characterised by a chronic, indolent, and relapsing course [10,11,12]. This indolent course may be the result of the release of neutrophil extracellular traps (NETs) triggered by farnesol, a crucial quorum-sensing molecule of *C. albicans* [13]. The accumulation of NETs can reduce inflammation through the degeneration of cytokines and chemokines [14]. This could explain why *C. albicans*-infected CP-implant mice stopped limping before the noninfected CP-implant animals. In the CP + A-implant groups, limping decreased faster in *C. albicans*-infected mice compared to noninfected mice. This finding is uncertain but could be attributed to the indolent course provoked by the presence of *C. albicans* on the implant, although these yeasts are not viable. Piloerection did not show a clear difference between noninfected and Cal35-infected mice in non-coated or coated implants over time, contrasting with other bacterial PJI in vivo models [30]. The survival of our animal model varied according to the group. One of 11 mice from the Cal35-infected CP-implant group died as a result of renal “fungus balls” caused by *C. albicans* infection [18,19]. These balls are most likely the result of haematogenous seeding from septic arthritis of the knee joint [20]. This highlights the high mortality associated with candidemia derived from *Candida* bone and joint diseases [21,22,31]. However, these results must be interpreted cautiously, particularly when there is a difference of only one individual. Likewise, one of the 11 mice from the noninfected CP + A-implant group perished due to an acute hepato-digestive infection caused by mouse cytomegalovirus. This virus can be latent in different organs (e.g., liver) in immunocompetent mice, and cause acute infection in immunodeficient ones [32]. Furthermore, this virus can be detected as Grocott-positive intranuclear inclusions in pathological samples [33].

The microbiological and histological results obtained in this study revealed the difficulty of inducing this type of infection despite having used two of the most important pharmacological risk factors, i.e., immunosuppression [34] and broad-spectrum antibiotic therapy [35]. This fact could underline the importance of other risk factors, such as systemic disease, diabetes mellitus, revision arthroplasty, type of prosthesis (monoblock or modular), and type of fixation (uncemented, cemented, hybrid, or with plain or antibiotic-loaded cement) [27,28,29,31,34]. The most important finding of this work is that anidulafungin-loaded sol-gel coating, when applied to orthopaedic implants, can prevent Candida PJI in an in vivo model. Interestingly, some kind of osteomyelitis was detected in three mice, though no presence of yeast was observed. This finding may be due to both the presence of dead yeast killed by anidulafungin and the inhibition of yeast phagocytosis that dexamethasone therapy exerts on phagocytes [36], thereby explaining the inflammation in absence of yeast proliferation. Our results are consistent with other previously published in vitro studies [25]. Moreover, anidulafungin-loaded coating had a non-harmful effect on bone mineralisation according to the microcomputed tomographic images, and no changes in bone markers were found among groups, thus supporting the results obtained in previous research, based on sol-gel processes [30]. Hence, the fixation of an anidulafungin-loaded sol-gel coated implant is likely to be at least as effective as an uncoated implant.

In recent years, several types of coating have been presented for clinical use: natural, peptide, ceramic, and synthetic coatings [37,38]. Most were designed for osteointegration and antibacterial purposes [30,39,40,41,42]. To our knowledge, few studies have developed these coatings to be loaded with antifungals associated with sol-gel technology, as proposed in this work [25]. As fungal PJI prediction is difficult, the use of anidulafungin-loaded sol-gel may be recommended in those patients who have risk factors for developing fungal PJI [35]. This would reduce the personal and healthcare costs associated with this type of infection and its relapses following delayed reimplantation arthroplasty after a follow-up of more than 50 months [43].

However, this study is not exempt from limitations. Firstly, the form of implant infection may have reduced yeast viability on anidulafungin-loaded sol-gel before implantation. No alternative form of infection was possible according to the results obtained in pilot studies (data not shown). Secondly, the results would be more robust with an equal number of samples allocated for microbiological and pathological analyses, although our number of specimens is nearly double that of similar, recently published studies [30]. This unexpected limitation stems from the low infectivity shown by *C. albicans* (approximately 50%), as evidenced in this study. Thirdly, this type of technology can carry other antifungals, e.g., fluconazole [22], which provides it with an antifungal ability against both *C. albicans* and some non-*C. albicans* species, and which should be evaluated using in vivo models for preventive use in some special cases. Fourthly, the death caused by cytomegalovirus suggests that this animal model should be replicated in an Animal Biosafety Level-2 facility, where moderately immunosuppressed animals are less exposed to environmental pathogens.

## 4. Materials and Methods

### 4.1. Sol-Gel Synthesis and Coating of Titanium Implants

The Ti-6Al-4V implants were made from 0.6-mm thick Kirschner wires provided by Depuy Synthes (Johnson & Johnson, New Brunswick, NJ, USA). Each wire was cut into implants measuring 1 cm in length. Subsequently, these were chemically polished (CP), as previously described [44], to achieve a surface finish more closely resembling that used in routine clinical practice.

Hybrid organic–inorganic sol-gel coatings composed of a mixture of organopolysyloxanes, including methacryloxypropyltrimethoxy silane (MAPTMS, 98%, Acros Organics, Thermo Fisher Scientific, Waltham, MA, USA) and tetramethyl orthosilane (TMOS, 98%, Acros Organics, Thermo Fisher Scientific, Waltham, MA, USA) and biofunctionalised with tris(trimethylsilyl)phosphite (92%, Sigma-Aldrich, St. Louis, MO, USA) were prepared following a previously published methodology [23]. The coating was loaded with 0.99 mg/mL of anidulafungin (Pfizer, New York, NY, USA) by adding the drug to the aqueous phase during its preparation [22]. Finally, the Ti-6Al-4V implants for the in vivo model were coated by dipping them in anidulafungin and allowing them to dry for at least 1 h at 60 °C (CP + A).

### 4.2. Animal Surgical Model and Monitoring

**Figure 9:**
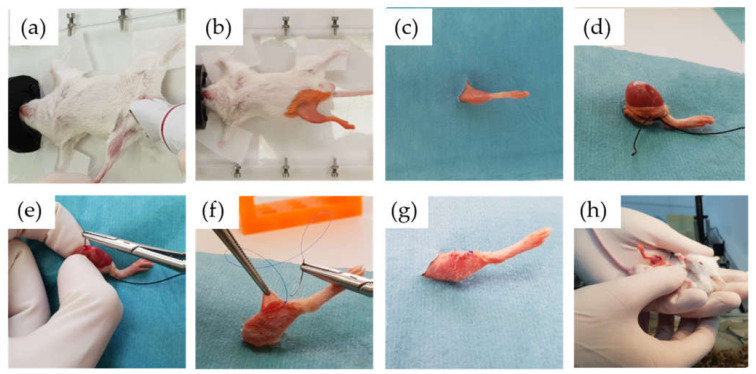
Surgical procedure: inhaled anaesthesia and shaving of the limb (**a**), antiseptic washing and isolation of the surgical field (**b**,**c**), skin dissection and exposure of the bony entry point (**d**), retrograde introduction of the biomaterial into the femur of the mouse (**e**), suture and cleaning of the surgical wound (**f**,**g**), awakening and care of the animal (**h**).

**Figure 10:**
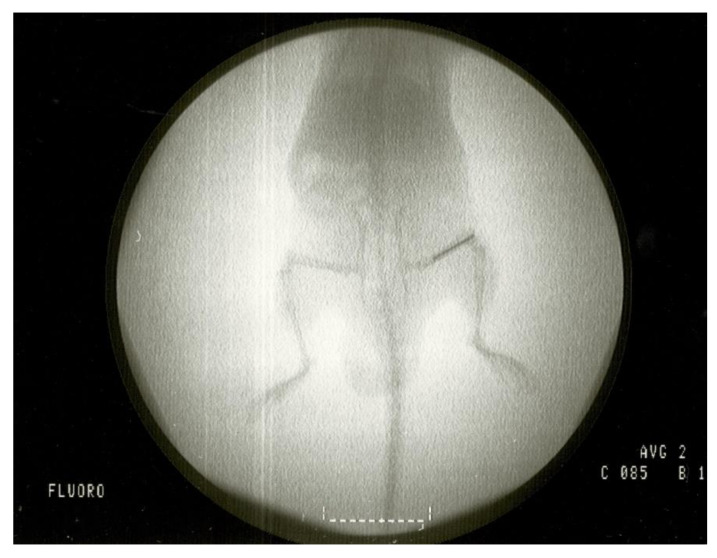
Fluoroscopy of a mouse with an implant placed in the femur.

We used one clinical strain isolated in the clinical microbiology department of Fundación Jiménez Díaz University Hospital: a strain of *C. albicans* from an 81-year-old woman with infection of a hip prosthesis (Cal35). The antifungal susceptibility profile of Cal35 was obtained by the Vitek 2 AST-YS08 yeast susceptibility test (bioMérieux, Marcy l’Etoile, France). Cal35 was susceptible to all antifungals tested, i.e., amphotericin B (≤0.25 µg/mL), caspofungin (≤0.25 µg/mL), flucytosine (≤1 µg/mL), fluconazole (2 µg/mL), micafungin (≤0.06 µg/mL), and voriconazole (≤0.12 µg/mL). Surgical intervention of the in vivo model was based on a modified model previously described by Aguilera-Correa et al. [30]. The intervention consisted of placing the implant into the right femur of RjOrl:SWISS (CD1) mice (Janvier Labs, France) through the knee using an aseptic surgical technique (Figure 9 and Figure 10). Two main modifications were made. Firstly, all mice were premedicated with 4 mg/L of dexamethasone [45] (B.Braun, Melsungen, Germany) and 0.1 mg/L of enrofloxacin (ganadexil 5%, Industrial Veterinaria, S.A.—Invesa, Spain) [46] in sterile drinking water one week before surgery and for the entire duration of the study. Secondly, the implant infection procedure consisted of incubating 1 mL of a 2.00 McFarland standard of Cal-35 strain in saline (B.Braun, Melsungen, Germany) with each implant in a 12-well plate at 37 °C and 5% CO_2_ for 120 min. After incubation, each implant was rinsed two times in saline. This form of implant infection aims to adhere the yeast to the surface of the implant, due to the impossibility of injecting planktonic yeasts into the femur. In pilot studies conducted prior to this, in vivo model animals infected by planktonic yeasts in the femur before implantation of the prosthesis died from *Candida* infections associated with the liver, kidneys, or lungs (data not shown).

Sixteen-week-old male mice with femoral implants were randomly distributed into four groups: one group with a CP implant without infection (CP group, *n* = 11), a group with a CP implant with infection induced by Cal35 (CP Cal35 group, *n* = 11), the third with a CP implant coated with anidulafungin-loaded sol-gel without infection (CP + A group, *n* = 11), and the fourth with a CP implant coated with anidulafungin-loaded sol-gel with infection induced by Cal35 (CP + A Cal35 group, *n* = 11). The sample size was estimated by Wilcoxon Mann–Whitney test and an a priori type of power analysis, considering *d* = 1.5, α = 0.05, (1-β) = 0.95, allocation ratio = 1 by using G*Power 3.1.9.7 software [47]. The *d* parameter is based on the assumption that the anidulafungin-loaded coating is able to reduce the yeast concentration by at least 80% per gram of bone when compared to the uncoated implant group. The statistical power of the sample was 0.9522. All the animals were included in the study and there were no exclusions.

We assessed the pain-stress and weight of each animal every 48 h on weekdays to ensure physical status. Evaluation of pain/stress was based on the presence or absence of six directly related behaviours in this species for the surgical procedure the animals underwent, i.e., limping, piloerection, lack of grooming, wound presence, passivity, and aggressiveness. In cases of sustained weight loss over time, the most appropriate refinement measures were taken to encourage the animal to eat. For this, they were offered an additional mixture of grains and vegetables (Vitakraft, Bremen, Germany). Five weeks (35 days) after surgery, all the animals were euthanised using hypercapnia. The right femur of each animal was then removed following sterile preparation of the knee, and the samples were sent for analysis. In case of the pre-euthanasia death of one of the mice in any group, the operated femur was alternatively used for microbiological or pathological studies.

### 4.3. Microbiological and Pathological Studies

After euthanasia and previous extraction of the femurs from Cal35-infected mice, joint fluid samples were taken using sterile swabs, and this fluid was used to make stamps on a slide for Gram staining. The 11 mice in the Cal-35-infected group were divided into two subgroups: 6 animals were used for microbiological studies and 5 for pathological studies. Three femurs from each noninfected group were used for microbiological studies.

Extracted bones were processed according to the methodology previously described by Aguilera-Correa et al. [30]. Briefly, using a hammer, each femur was divided into two samples in a sterile bag: (1) bone and adnexa and (2) implant. The bone was immersed in 2 mL of saline and sonicated using a sonicator at 22 °C for 5 min [48]. The resulting sonicate was diluted in a 10-fold dilution bank and seeded on chloramphenicol-gentamicin Sabouraud agar (bioMérieux, Marcy l’Etoile, France) using the plaque extension method, which consists of seeding 100 µL/plate of each dilution. The concentration of yeasts was estimated as CFU/g of bone and adnexa. The implant was sonicated in 2 mL of saline for 5 min to release the adhered yeast biofilm and to estimate biofilm concentration, measured as CFU/cm^2^ of the implant. All plates were checked at 48 and 72 h.

The five femurs obtained from the Cal35-infected group were fixed in 4% paraformaldehyde for 48 h, decalcified in 10% ethylenediaminetetraacetic acid (EDTA) for 4 weeks, paraffin-infiltrated, and stained with haematoxylin-eosin. The presence of some necrotic trabecular bone and some repair areas, fibrosis, and adipose replacement of the bone marrow were identified and recorded. The presence of yeast was determined by using Grocott’s silver stain [49]. The presence/absence of round or ovoid structures, with or without signs of germination, was recorded.

Histopathological definitions were as follows:

- Acute osteomyelitis was defined as bone tissue evidencing moderate-to-high–intensity polymorphonuclear (PMN) inflammatory response with tissue necrosis phenomena and trapping of trabecular bone remains;
- Chronic osteomyelitis was defined as bone tissue that presents a variable inflammatory reaction, partially consisting of a PMN response, but mainly of plasma cells and lymphocytes;
- PJI was diagnosed when any type of osteomyelitis and the presence of yeast were evidenced.

### 4.4. Microcomputed Tomography

Eight bone samples from each noninfected group included in the aforementioned model were fixed in 10% formaldehyde for 48 h at 4 °C. After fixation, the samples were dehydrated in 96% ethanol for 48 h, changing the ethanol every 24 h, and in 100% ethanol for 48 h, changing the ethanol every 24 h. Hind legs were removed and fixed in 10% neutral buffered formalin. Before CT scanning, the paws were washed with running water for 15 min. Three-dimensional microcomputed tomographic imaging was performed with a CompaCT scanner (SEDECAL Madrid, Spain). Data were acquired with 720 projections by 360° scan, the integration time of 100 ms with three frames, a photon energy of 50 KeV, and current of 100 uA. The duration of imaging time was 20 min per scan. Three-dimensional renderings of images of hind paws were generated through original volumetric reconstructed images by MicroView software (GE Healthcare, Boston, MA, USA). Comparable regions of interest consisting of three metatarsal joints from each mouse were selected for analysis. Bone volume (BV), bone mass (BM), BMD (calculated as BM/BV mg/cm^3^), and mean cortical thickness (mm) were quantified from micro-CT scans using GE MicroView software v2.2.

### 4.5. Immunohistochemistry

Five out of eight femurs used for microcomputed tomography from each group were decalcified in 10% EDTA for 4 weeks, paraffin-infiltrated, and stained with haematoxylin-eosin. In the noninfected groups, implants were removed and transversal sections in the knee condyles (5 µm) were made. Immunohistochemical analysis was carried out as previously described [50]. Briefly, sections were incubated with proteinase K Solution (20 µg/mL in Tris-EDTA Buffer, pH 8.0) for 15 min in a water bath at 37 °C for antigen retrieval after deparaffinisation and re-hydration. The blocking of nonspecific binding was performed with phosphate buffer saline (PBS), 3% bovine serum albumin (BSA) and 0.1% Triton X-100 for 1 h, and the primary antibodies anti-cathepsin K (1:25), cluster of differentiation 68 (CD68) (1:200), and alkaline phosphatase (ALP) 1:200 (all antibodies from Santa Cruz Biotechnology, Santa Cruz, CA, USA) were incubated overnight at 4 °C in a humidifying chamber. The secondary antibodies goat anti-rabbit-fluorescein isothiocyanate (FITC) (1:200) and goat anti-mouse FITC (1:200) (Invitrogen, Life Technologies, Carlsbad, CA, USA) were incubated for 1 h in the dark. Slides were mounted with Fluoroshield with 4′,6-diamidino-2-fenilindol (DAPI) mounting media (Sigma-Aldrich, St. Louis, MO, USA). Images were taken with the iScan Coreo Au scanner (Ventana Medical Systems, Roche Diagnostics, Basel, Switzerland) and visualised with Image Viewer v.3.1 software (Ventana Medical Systems, Roche Diagnostics, Basel, Switzerland). Images were taken at 4× or 10× magnification.

### 4.6. Statistical Analysis

The primary hypotheses were such that the anidulafungin-loaded sol-gel would prevent *C. albicans* PJI and that the anidulafungin-loaded sol-gel can be used without altering bone metabolism at any level. The secondary hypothesis was that the effect of anidulafungin-loaded sol-gel in preventing PJI could be evaluated by animal monitoring before euthanasia.

Statistical analyses were performed using Stata Statistical Software, Release 11 (StataCorp, College Station, TX, USA). Data were evaluated using a one-sided Wilcoxon non-parametric test to compare 2 groups or a one-sided proportion comparison Z-test. A log-rank test was used to perform a pairwise comparison of the Kaplan–Meier survival curves of two groups. Statistical significance was set at *p* ≤ 0.05. Body weight was evaluated over time using a linear regression model. Microbiological results and weight values are represented as median and interquartile range. Other behavioural variables are represented as relative frequencies at each time point.

## 5. Conclusions

In conclusion, anidulafungin-loaded sol-gel can prevent PJI caused by *C. albicans* without compromising bone integrity.

## 6. Patents

The sol-gel used in this study is one of the products protected by the Spanish patent system with Publication Number 2686890, applied for 19 April 2017, and entitled Procedure for Obtaining a Sol-Gel Coating, Composition Coating and Use of the Same.

## Data Availability

Data supporting reported results can be found by contacting with the corresponding authors.
